# Hydrophobicity-Driven Increases in Editing in Mitochondrial mRNAs during the Evolution of Kinetoplastids

**DOI:** 10.1093/molbev/msad081

**Published:** 2023-04-08

**Authors:** Fanny Rusman, Noelia Floridia-Yapur, Anahí G Díaz, Tatiana Ponce, Patricio Diosque, Nicolás Tomasini

**Affiliations:** Unidad de Epidemiología Molecular, Instituto de Patología Experimental, Universidad Nacional de Salta-CONICET, Salta, Argentina; Unidad de Epidemiología Molecular, Instituto de Patología Experimental, Universidad Nacional de Salta-CONICET, Salta, Argentina; Unidad de Epidemiología Molecular, Instituto de Patología Experimental, Universidad Nacional de Salta-CONICET, Salta, Argentina; Unidad de Epidemiología Molecular, Instituto de Patología Experimental, Universidad Nacional de Salta-CONICET, Salta, Argentina; Unidad de Epidemiología Molecular, Instituto de Patología Experimental, Universidad Nacional de Salta-CONICET, Salta, Argentina; Unidad de Epidemiología Molecular, Instituto de Patología Experimental, Universidad Nacional de Salta-CONICET, Salta, Argentina

**Keywords:** kinetoplastids, RNA editing, pan-editing, hydrophobic ratchet

## Abstract

Kinetoplastids are a diverse group of flagellates which exhibit editing by insertion/deletion of Us in the mitochondrial mRNAs. Some mRNAs require editing to build most of their coding sequences, a process known as pan-editing. Evidence suggests that pan-editing is an ancestral feature in kinetoplastids. Here, we investigate how the transition from nonedited to pan-edited states occurred. The mitochondrial mRNAs and protein sequences from nine kinetoplastids and related groups (diplonemids, euglenids, and jakobids) were analyzed. RNA editing increased protein hydrophobicity to extreme values by introducing Us in the second codon position, despite the absence of editing preferences related to codon position. In addition, hydrophobicity was maintained by purifying selection in species that lost editing by retroposition of the fully edited mRNA. Only a few hydrophobic to hydrophilic amino acid changes were inferred for such species. In the protein secondary structure, these changes occurred spatially close to other hydrophilic residues. The analysis of coevolving sites showed that multiple changes are required together for hydrophobicity to be lost, which suggest the proteins are locked into extended hydrophobicity. Finally, an analysis of the NAD7 protein–protein interactions showed they can also influence hydrophobicity increase in the protein and where editing can occur in the mRNA. In conclusion, our results suggest that protein hydrophobicity has influenced editing site selection and how editing expanded in mRNAs. In effect, the hydrophobicity increase was entrenched by a neutral ratchet moved by a mutational pressure to introduce Us, thus helping to explain both RNA editing increase and, possibly, persistence.

## Introduction

Kinetoplastids are a clade of flagellate protozoa that includes species with medical, veterinary, and/or ecological importance. The most studied genera in the clade are *Leishmania* and *Trypanosoma*, with several species causing neglected diseases: leishmaniasis, Chagas disease, and sleeping sickness. In addition, kinetoplastids have unique features among eukaryotes. One of many peculiarities is a single mitochondrion with a complex network of concatenated DNA rings (Cavalcanti and de Souza 2018). Maxicircles are the DNA rings that code for mitochondrial ribosomal RNAs (9*S* and 12*S*) and <20 mitochondrial proteins (Simpson et al. 1987; Ruvalcaba-Trejo and Sturm 2011; Lin et al. 2015). Several of such genes require posttranscriptional modifications in their pre-mRNA to generate functional open-reading frames. The process is known as mRNA editing, and it is made by inserting (most commonly) or deleting uridines (Us) (Simpson et al. 1996). Some genes are known as cryptogenes because their mRNAs require extensive editing, a phenomenon known as pan-editing (Feagin et al. 1988). In addition, pan-editing is an ancestral feature of kinetoplastids that was lost in different lineages and genes (Gray 1994; Maslov et al. 1994; Simpson and Maslov 1994a). Some clades phylogenetically close to kinetoplastids, such as diplonemids and euglenids, have no evidence of current or past editing by inserting or deleting Us (Dobakova et al. 2015; Burger and Valach 2018). Consequently, it is unknown how this transition from nonediting to pan-editing has occurred in kinetoplastids.

U insertion/deletion requires complex and energetically expensive machinery and thousands of DNA minicircles coding short RNAs called guide RNAs (gRNAs), which indicate where editing must occur (Estevez and Simpson 1999; Simpson et al. 2003; Read et al. 2016; Rusman et al. 2021). Different evolutionary hypotheses have been proposed to explain the origin and persistence of this expensive editing system, although no consensus has been achieved yet (Gray et al. 2010; Speijer 2010; Flegontov et al. 2011). Gray et al. proposed several years ago that editing in kinetoplastids is an example of constructive neutral evolution (CNE) (Covello and Gray 1993; Gray et al. 2010; Flegontov et al. 2011). In this framework, editing has no adaptive advantages, and kinetoplastids were trapped in a ratchet of complexity increase. Such a ratchet would require that the editing process not be lost by a single mutation step; otherwise selection pressures would favor cheaper systems without editing. The main criticism to the hypothesis of editing in kinetoplastids as an example of CNE was that editing can be lost in a single mutational step, that is, the replacement of the cryptogen in the maxicircle by a retrotranscribed fully edited mRNA (Speijer 2010). Despite this, CNE should not be discarded if the appearance of novel editing functions is considered or selective pressures for cheaper systems are not so strong (Lukes et al. 2021). Recently, it has been proposed that CNE can explain the origin of multimeric proteins by a hydrophobic ratchet (Hochberg et al. 2020). Basically, mutations at the binding interface between monomers accumulate hydrophobic amino acids by mutation. Such mutations are neutral in the dimeric or multimeric state since such sites stay buried and hidden from the solvent. However, they are deleterious in the monomeric state when such sites are exposed. Consequently, mutations that change hydrophilic to hydrophobic amino acids at the protein surface may favor multimerization. Purifying selection then entrenches the multimeric form because monomeric ones are now deleterious. Such a ratchet works even if there is no functional advantage for the multimeric form in comparison with the monomeric one. Interestingly, in kinetoplastids, the inserted Us probably contribute to increasing U content. Such an increase may affect protein hydrophobicity since the genetic code determines that codons with U residues in the second positions always code for hydrophobic amino acids. However, to our knowledge, the impact of the mRNA editing process on protein hydrophobicity and a potential hydrophobic ratchet were not analyzed at all.

In this paper, we address the evolutionary transition from nonedited (ancestral state) to pan-edited mRNAs. For that purpose, we analyzed available sequences of mitochondrion-encoded mRNA in kinetoplastids, their related groups within Euglenozoa (diplonemids and euglenids), and in a clade related to Euglenozoa (jakobids). In addition, we investigated how the hydrophobicity of the proteins has driven this transition—from nonedited mRNAs to pan-edited ones—and how this transition generated an extreme hydrophobicity increase in the proteins. Finally, we discuss the implications of our results for the neutral and adaptationist hypotheses about mRNA editing in kinetoplastids.

## Results

### Kinetoplastids Have an Increased U Content in Mitochondrial Pan-Edited mRNAs with No Preferential U Distribution on Codon Positions

**Fig. 1.**
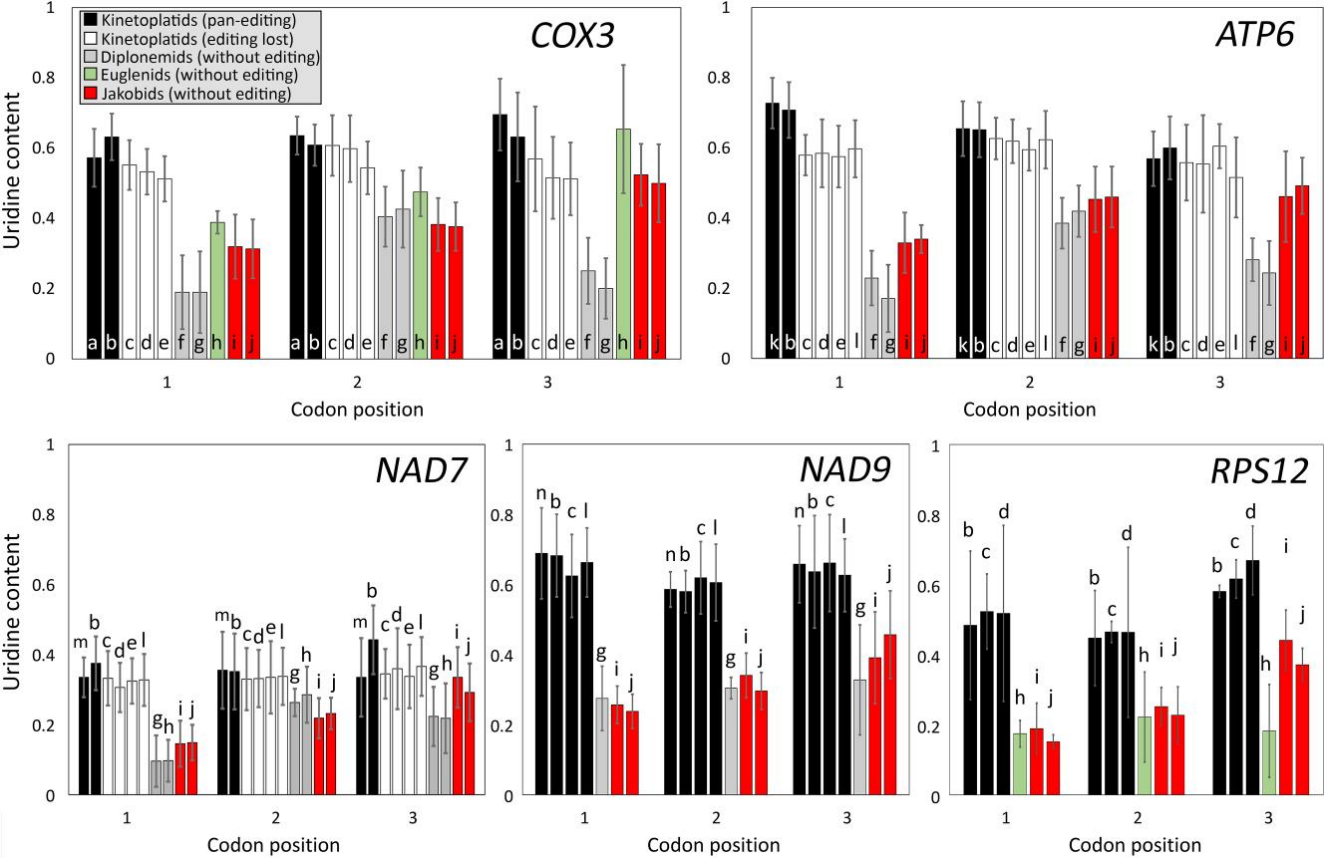
U content at different codon positions for five pan-edited genes in kinetoplastids and their homologs in diplonemids, euglenids, and jakobids. The U content at each codon position is calculated as the proportion of sites in that position that contain U nucleotides. Black bars indicate kinetoplastid species with pan-editing for the gene; white bars indicate kinetoplastid species with no or partial editing; gray bars indicate diplonemid species; green bars indicate euglenids and red bars indicate jakobid species. a, *Herpetomonas muscarum*; b, *T. brucei*; c, *L. tarentolae*; d, *C. fasciculata*; e, *A. deanei*; f, *D. ambulator*; g, *Lacrimia lanifica*; h, *E. gracilis*; i, *J. libera*; j, *R. americana*; k, *Trypanosoma cruzi*; l, *L. pyrrhocoris*; m, *Bodo saltans*; n, *Trypanosoma vivax*.

**Fig. 2.**
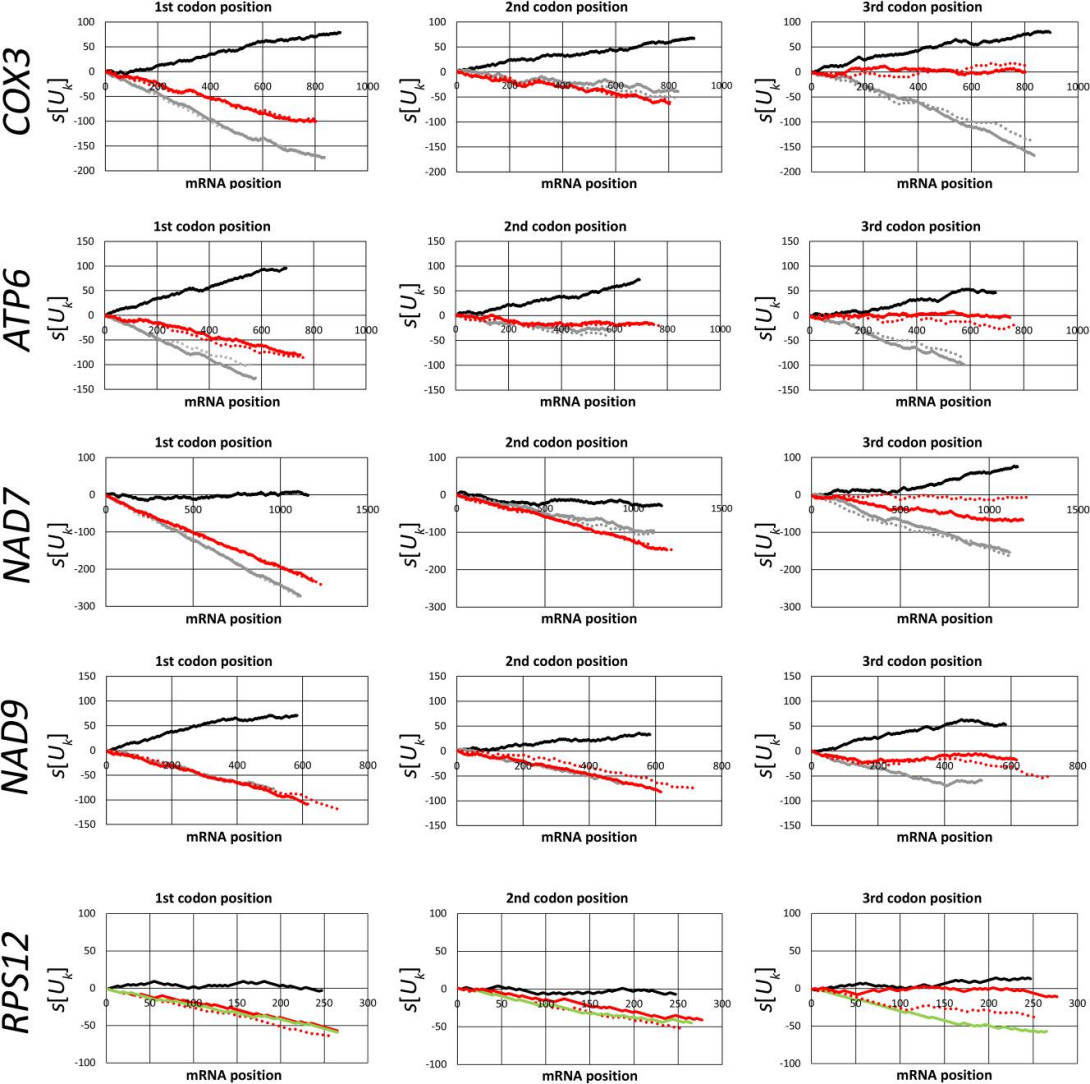
U walk at different codon positions for pan-edited mRNAs in *T. brucei* and mRNAs in diplonemids, euglenids, and jakobids. The *s*[*U_k_*] value increases one unit every time that a U is at the *k* mRNA position in a certain codon position (first, second, or third), and it decreases the same value when there is another base. A positive slope in a region of the line indicates there are more Us than other bases, whereas a negative one indicates the opposite. Regions with parallel lines indicate similar U content between species. Black line, *T. brucei*; solid red line, *J. libera*; dotted red line, *R. americana*; solid gray line, *D. ambulator*; dotted gray line, *Lacrimia lanifica*; green line, *E. gracilis*.

To determine how U insertion affected hydrophobicity in mitochondrion-encoded proteins, we first analyzed the U content in mitochondrial mRNAs in kinetoplastids and related groups, that is, diplonemids, euglenids, and jakobids (table 1) for different mitochondrial genes. We divided the analyzed genes into two groups. The first one includes genes that are pan-edited in trypanosomes but have lost pan-editing in some kinetoplastids (pan-edited genes). The second group includes genes that are nonedited or partially edited in all studied kinetoplastids (nonedited or partially edited genes). On average, the U content was higher in kinetoplastids than that in the related groups. This higher content in kinetoplastids was observed in both pan-edited genes (*COX3*, *NAD7*, *NAD9*, *ATP6*, and *RPS12*) and nonedited or partially edited genes (table 1). Introducing Us at the second position of any codon results in a hydrophobic amino acid, although at the third position, it may be related to codon usage preference. Consequently, we first analyzed whether there is a differential distribution of the total Us at different codon positions (fig. 1 and supplementary fig. S1, Supplementary Material online). Considering pan-edited genes (*COX3*, *NAD7*, *NAD9*, *ATP6*, and *RPS12*), no differential distribution was observed in terms of codon positions for the total Us for any species (table 2 and fig. 1). This pattern was also observed at different regions in the sequence of such pan-edited mRNAs (fig. 2). Instead, *COX1* and *NAD1* (both nonedited) showed a differential distribution favoring Us at the second and third codon positions in kinetoplastids (table 2 and supplementary fig. S1, Supplementary Material online). In addition, the analyzed pan-edited genes in *Trypanosoma brucei* (the trypanosome with most complete information about editing) exhibit homogeneous distribution of inserted Us (table 3). Instead, a differential U distribution at codon positions was mainly observed for diplonemids, euglenids, and jakobids (table 2, fig. 1, and supplementary fig. S1, Supplementary Material online). For instance, *Jakoba libera* and *Reclinomonas americana* (jakobids) had lower U content in the first codon position in all analyzed genes (fig. 1 and supplementary fig. S1, Supplementary Material online). The same was observed for *COX1*, *COX2*, *COX3*, and *NAD5* in *Euglena gracilis* (euglenid) (supplementary fig. S1, Supplementary Material online). A higher U content in the second codon position was observed in diplonemids for most genes (7/8) (fig. 1 and supplementary fig. S1, Supplementary Material online). These results suggest that editing has increased the U content in the ancestor of kinetoplastids. In addition, such an increase had no preference for any codon position, which suggests that it was not directed by selective pressures related to codon position.

**Table 1.** U Content in Mitochondrion-Encoded mRNAs for Kinetoplastids, Diplonemids, Euglenids, and Jakobids.

	Kinetoplastids				Diplonemids		Euglenid	Jakobids	
	*T. brucei*	*L. tarentolae*	*C. fasciculata*	*A. deanei*	*D. ambulator*	*L. lanifica*	*E. gracilis*	*J. libera*	*R. americana*
*COX1*	0.48 (0.09)	0.48 (0.09)	0.45 (0.12)	0.47 (0.09)	0.29 (0.07) ***	0.28 (0.06) ***	0.45 (0.09)	0.38 (0.09) ***	0.39 (0.10) **
*COX2*	0.43 (0.09)	0.46 (0.09)	0.46 (0.09)	0.44 (0.1)	0.28 (0.06) ***	0.27 (0.07) ***	0.39 (0.10)	0.39 (0.08)	0.38 (0.09) *
*COX3*	**0.62 (0.07)**	0.58 (0.08) *	0.55 (0.08) **	0.52 (0.09) ***	0.28 (0.07)***	0.27 (0.07) ***	0.51 (0.09)^a^ ***	0.41 (0.09) ***	0.4 (0.09) ***
*CYTB*	0.48 (0.10)	0.5 (0.11)	0.51 (0.11)	na	0.28 (0.06) ***	0.28 (0.05) ***	na	0.43 (0.10) *	0.42 (0.11) **
*NAD1*	0.45 (0.09)	0.43 (0.12)	0.42 (0.10)	0.45 (0.14)	0.28 (0.10) ***	0.28 (0.06) ***	0.44 (0.13)	0.36 (0.09) **	0.34 (0.10) ***
*NAD5*	0.53 (0.11)	0.54 (0.10)	0.56 (0.12)	0.52 (0.11)	0.29 (0.10) ***	na	0.46 (0.09) ***	0.46 (0.13) **	0.48 (0.10) ***
*NAD7*	**0.39 (0.07)**	0.34 (0.08) **	0.33 (0.08) **	0.33 (0.09) **	0.19 (0.07) ***	0.2 (0.07) ***	na	0.23 (0.09) ***	0.22 (0.09) ***
*NAD9*	**0.63 (0.09)**	**0.63 (0.09)**	na	na	0.3 (0.05) ***	na	na	0.33 (0.11) ***	0.33 (0.1) ***
*ATP6*	**0.65 (0.11)**	0.59 (0.09) *	0.59 (0.09) *	0.59 (0.10) *	0.3 (0.05) ***	0.28 (0.06) ***	na	0.41 (0.09) ***	0.43 (0.08) ***
*RPS12*	**0.50 (0.17)**	**0.53 (0.13)**	**0.55 (0.10)**	na	na	na	0.19 (0.08) ***	0.29 (0.14) ***	0.25 (0.10) ***

Note.—Value in parentheses indicates standard deviation calculated over nonoverlapping segments of 30 bases. Bold values indicate pan-edited mRNAs. Underlined values indicate genes that require partial editing. A chi-square test was used to compare the proportion of Us from each species in relation to that of *T. brucei*. Statistical significance was considered at *P < 0.05; **P < 0.01; ***P < 0.001. na, sequence not available.

a Short sequence (484 bp).

**Table 2.** Chi-square Test for the Hypothesis of Homogeneous Distribution of Us at First, Second, and Third Codon Positions.

	Kinetoplastids				Diplonemids		Euglenid	Jakobids	
mRNA	*T. brucei*	*L. tarentolae*	*C. fasciculata*	*A. deanei*	*D. ambulator*	*L. lanifica*	*E. gracilis*	*J. libera*	*R. americana*
*COX1*	**0**.**0015**	**0**.**0028**	**0**.**0059**	**3.35 × 10^−5^**	**2 × 10^−9^**	**3 × 10^−9^**	**1 × 10^−7^**	**8.7 × 10^−8^**	**8 × 10^−7^**
*COX2*	0.26	0.81	0.64	0.47	**8 × 10^−4^**	**0.0011**	**0.0012**	**1.7 × 10^−8^**	**4 × 10^−5^**
*COX3* ^a^	0.18	0.67	0.36	0.84	**5 × 10^−6^**	**1 × 10^−8^**	**0.0028**	**7 × 10^−4^**	**0.0023**
*NAD1*	**0**.**0097**	**0**.**002**	**2 × 10^−4^**	**7 × 10^−5^**	**2 × 10^−5^**	**1 × 10^−5^**	0.48	**0.016**	**0.006**
*NAD5*	**0**.**036**	0.53	0.83	0.23	**0.0045**	**1 × 10^−6^**	*na*	**0.0026**	**9 × 10^−8^**
*NAD7* ^a^	0.11	0.92	0.43	0.95	**4 × 10^−7^**	**4 × 10^−8^**	*na*	**1 × 10^−7^**	**1 × 10^−4^**
*NAD9* ^b^	0.44	0.85	*na*	*na*	**0.017**	na	*na*	**0.036**	**3 × 10^−4^**
*ATP6* ^a^	0.36	0.61	0.66	0.92	**0.017**	**1 × 10^−5^**	*na*	**0.033**	**0.024**
*RPS12* ^b^	0.46	0.39	0.15	*na*	*na*	*na*	0.65	**6 × 10^−4^**	**0.001**

Note.—The values indicate the *P* value for a chi-square test with a null hypothesis of a homogeneous distribution of Us at the first, second, and third codon positions. Bold values indicate *P* < 0.05 discarding the hypothesis of homogeneous distribution of the U content in different codon positions showing preference for certain codon position. Note that pan-edited mRNAs have nonstatistically significant *P* values in kinetoplastids. *na*, not available.

a Pan-edited in *T. brucei* and partially edited in other kinetoplastids.

b Pan-edited in the analyzed kinetoplastids.

**Table 3.** Comparison of Inserted and Total Us at Different Codon Positions in Pan-Edited mRNAs of *T. brucei*.

	Codon position			
mRNA^a^	1	2	3	*P* value^d^
*COX3*	171^b^/189^c^	168/182	173/189	0.96
*NAD7*	130/145	122/136	142/171	0.46
*NAD9*	121/133	105/113	109/124	0.54
*ATP6*	149/164	141/151	123/139	0.28
*RPS12*	35/40	34/37	43/48	0.52
*All*	606/671	570/619	590/671	0.58

a Only the CDS in the mRNA was considered.

b Inserted Us by mRNA editing.

c Whole Us in the CDS after editing.

d Chi-square test of homogeneity of inserted Us.

### Kinetoplastids Have Increased Hydrophobicity Indexes and Reduced Frequency of Disorder-Promoting Amino Acids in Proteins Coded by Pan-Edited Genes

**Fig. 3.**
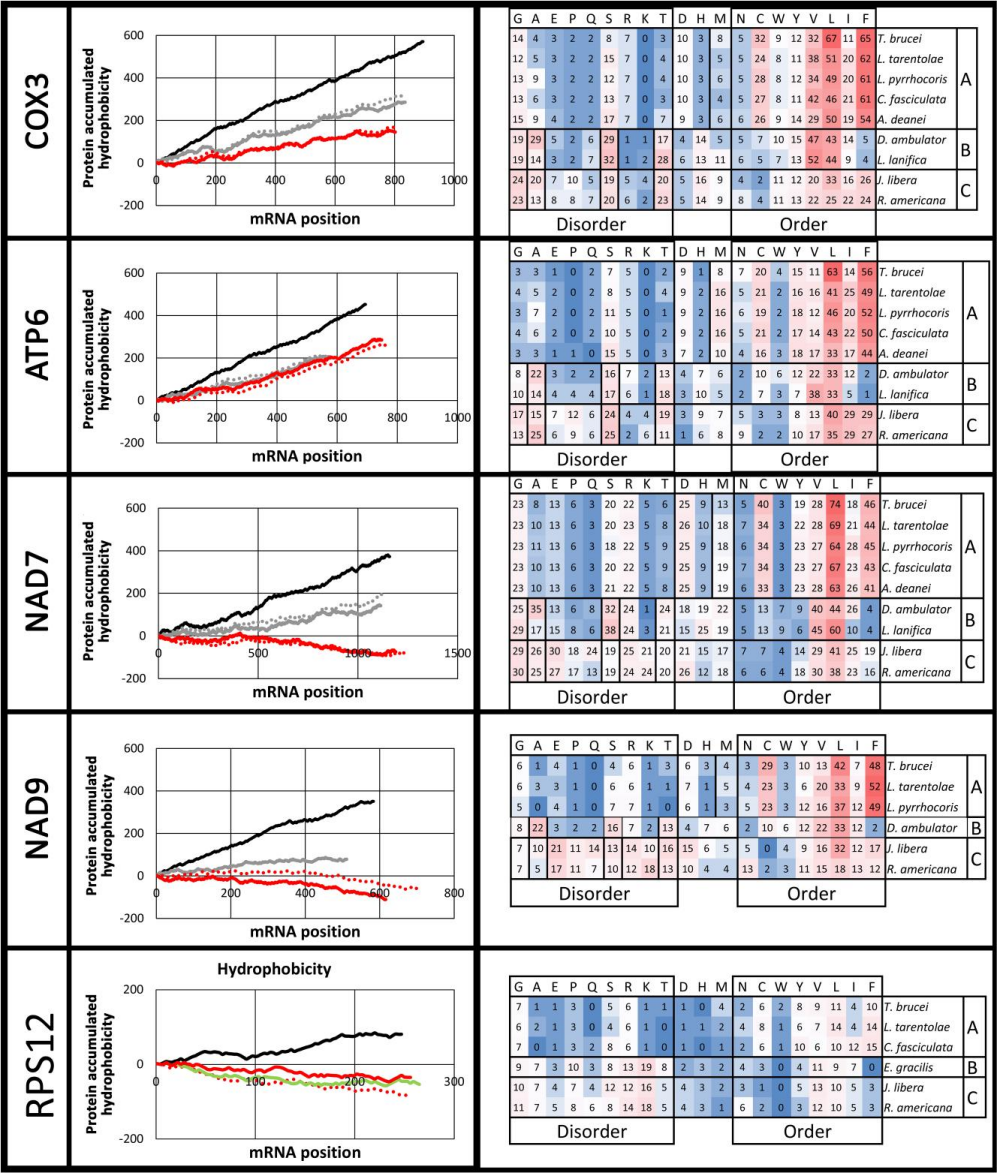
Changes in hydrophobicity and frequency of order- and disorder-promoting amino acids. (Left) Cumulative Kyte–Doolittle index of hydrophobicity (Kyte and Doolittle 1982) along the protein according to the mRNA position for pan-edited genes in *T. brucei* (black line) and their nonedited homologs in diplonemids (gray lines) and jakobids (red lines). Solid gray line, *D. ambulator*; dotted gray line, *Lacrimia lanifica*; solid red line, *J. libera*; dotted red line, *R. americana*. (Right) Order-/disorder-promoting amino acids composition for the different proteins. Colors indicate the frequency of such amino acids on a scale of 0 (blue) to the median (white) to the maximum value (red). A, kinetoplastids; B, diplonemids; C, jakobids.

Considering that U insertion at the second codon position introduces a hydrophobic amino acid, we compared hydrophobicity levels in proteins coded by mitochondrial pan-edited mRNAs from *T. brucei* against nonedited homologs in diplonemids or euglenids and jakobids. As expected, we observed a higher accumulated hydrophobicity index for all proteins coded by pan-edited mRNAs (fig. 3, left). In addition, differences in amino acid composition were observed. The most frequent amino acids in *T. brucei* for cytochrome c oxidase subunit III (COX3) and ATP synthase subunit 6 (ATP6), NAD7, RPS12, and NAD9 were leucine (L), phenylalanine (F), valine (V), and cysteine (C), which are order-promoting residues (i.e., the first three are common in secondary structures, and C forms disulfide bonds). Instead, diplonemids and jakobids had a higher frequency of threonine (T) and disorder-promoting amino acids such as serine (S) and alanine (A) (fig. 3, right).

**Fig. 4.**
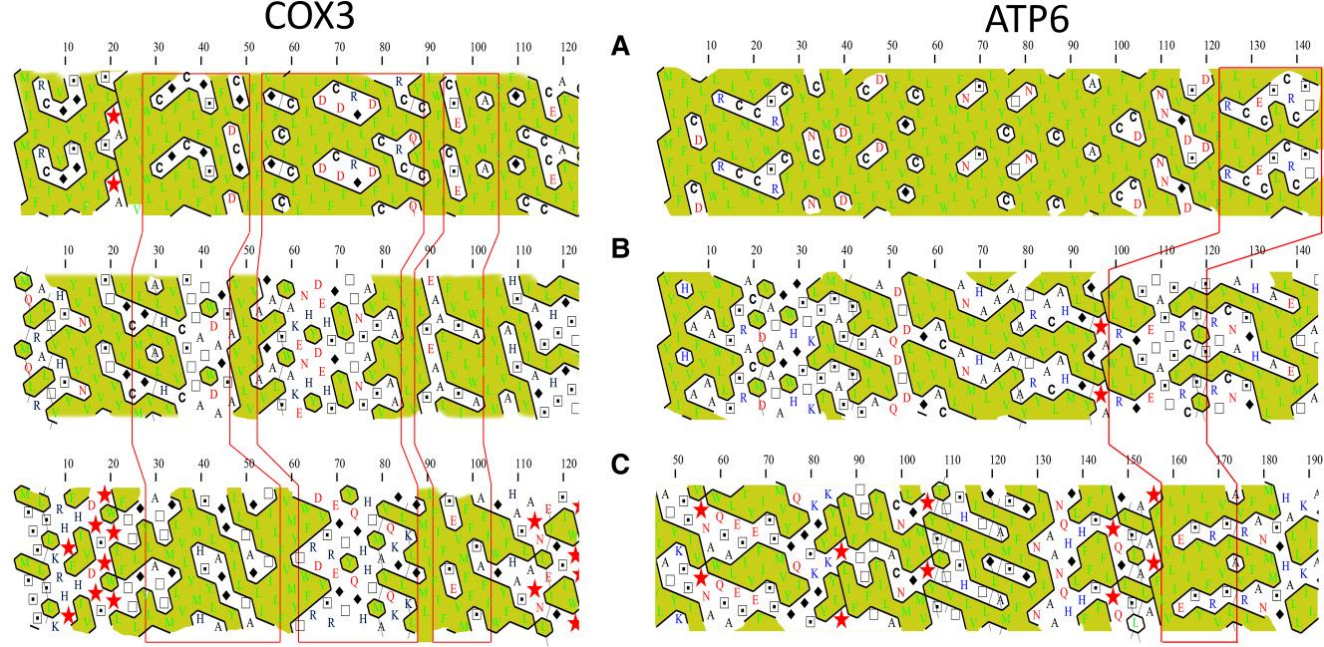
Reduced hydrophilic regions in *T. brucei* cytochrome c oxidase subunit 3 (COX3) and ATP synthase subunit 6 (ATP6) estimated by HCA. A, kinetoplastid (*T. brucei*); B, diplonemids (*D. ambulator*); C, jakobids (left, *J. libera*; right, *R. americana*). The primary amino acid sequence is two-dimensionally written on a duplicated alpha-helical net displaying adjacent amino acids that might be near each other if they were found in an alpha-helix. Only N-terminal fragments for COX3 and ATP6 are shown. Hydrophobic amino acids are shown in green. The hydrophobic clusters are delimited with black lines and colored (yellow). Special symbols are used for other amino acids: stars for prolines, squares, and dotted squares for threonines and serines and diamonds for glycines. Blue letters represent basic amino acids (R, K, and H); red letters indicate the acidic amino acids (D and E) and their uncharged counterparts (Q and N). Regions delimited by red lines indicate regions with potential homology (based on acidic or basic amino acid composition).

Considering the higher frequency of hydrophobic (and most order-promoting) amino acids, we made a hydrophobic cluster analysis (HCA) to address how such differences affect secondary structures in the kinetoplastid proteins. HCA is based on a duplicated 2D representation of the protein sequence, which highlights local proximities between amino acids in secondary structures (Woodcock et al. 1992). The HCA plots of COX3 and ATP6 N-terminals for *T. brucei* against *J. libera* and *Diplonema ambulator* are shown in figure 4. It is observed for *T. brucei* that hydrophilic regions are smaller than those observed in jakobids and diplonemids. Interestingly, most hydrophobic clusters were merged resulting in a pattern of hydrophilic clusters in a hydrophobic background in this kinetoplastid. Similar results were observed for NAD7, NAD9, and other regions of COX3 and ATP6 (supplementary figs. S2–S4, Supplementary Material online). These results show that pan-editing extended hydrophobic regions in the protein. Despite such changes, the predicted 3D structures of COX3 for *T. brucei and D. ambulator* show conserved structures mainly composed of six long alpha-helices, four of them packed tightly and two more relaxed (supplementary fig. S5, Supplementary Material online). In addition, surface hydrophobicity is increased in *T. brucei* against *D. ambulator*. Similar results were observed for NAD7 (supplementary fig. S5, Supplementary Material online).

### Extreme Protein Hydrophobicity of the Pan-Edited COX3

**Fig. 5.**
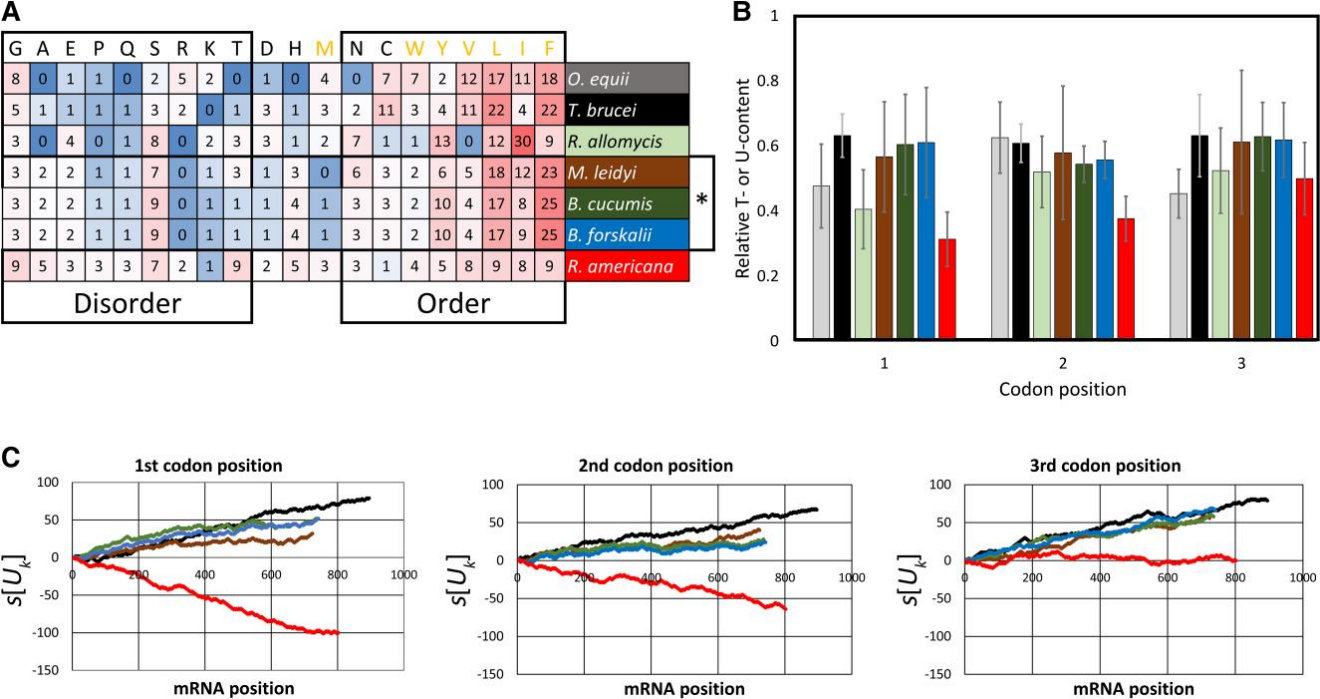
Eukaryotes with the highest frequency of order promoting and hydrophobic amino acids compared with *T. brucei.* (*A*) Amino acid composition of COX3 for the top-ranked species with the highest content of hydrophobic amino acids (yellow letters) compared with *R. americana* (jakobid). Asterisk indicates ctenophores. The analyzed species were *Oxyuris equi* (nematode), *Rozella allomycis* (fungi), *Mnemiopsis leidyi* (ctenophora), *Beroe cucumis* (ctenophora), and *Beroe forskalii* (ctenophora). (*B*) T or U content for different codon positions (same color references as in *A*). (*C*) U walks for COX3 in ctenophores show a similar pattern to *T. brucei* (black line) and different to *R. americana* (red).

Additionally, a comparison of 12,325 protein sequences retrieved from GenBank corresponding to mitochondrion-encoded COX3 from animals, plants, fungi, algae, and other protists (supplementary material S2, Supplementary Material online) showed that *T. brucei* is among the organisms with the highest content of hydrophobic amino acids (F, L, I, V, M, W, and Y) (supplementary material S2, Supplementary Material online and fig. 5*A*). The average proportion of hydrophobic amino acids for the data set was 0.48 ± 0.03, and such proportion was 0.68 for *T. brucei.* In addition, *T. brucei* COX3 was ranked as the second protein with the largest number of hydrophobic amino acids. Interestingly, among the organisms with the most hydrophobic COX3 is the clade of ctenophores. These metazoans have fast-evolving genes in the mitochondria (Pett et al. 2011; Kohn et al. 2012; Wang and Cheng 2019), and they probably have high levels of mutational bias. The analysis of the proportion of Ts in different codon positions and the T walks (Berger et al. 2004; see Materials and Methods) clearly show that COX3 in ctenophores has a mutational bias to higher T content with patterns of positive T walks similar to kinetoplastids (fig. 5*B* and *C*). The ranking of *T. brucei* COX3, as one of the most hydrophobic ones among other eukaryotes, suggests that the high hydrophobicity of this protein, although possible, is poorly explained by common substitution rates and selection. Instead, a high mutational rate with a T bias or an editing system that inserts Us (both may be considered mutational pressures) may reach such hydrophobicity.

### Hydrophobicity Loss Is Prevented by Purifying Selection after Missing mRNA Editing

**Fig. 6.**
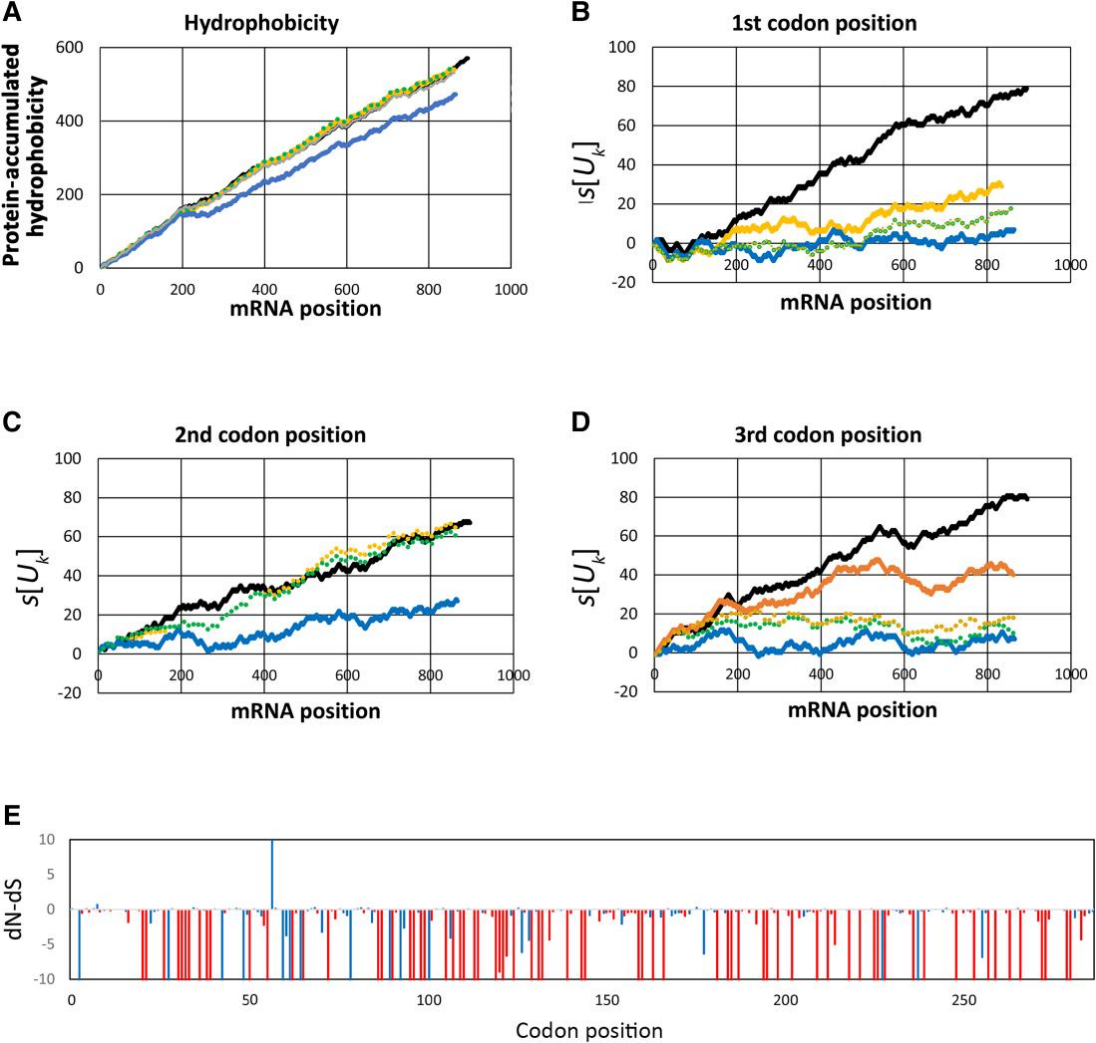
Purifying selection conserved hydrophobicity and U content at the second codon position after losing mRNA editing in *COX3.* (*A*) Cumulative hydrophobicity index based on the Kyte–Doolittle index of hydrophobicity. Black, *T. brucei*; solid orange, *L. tarentolae*; dotted orange, *Leptomonas pyrrhocoris*; dotted green, *C. fasciculata*; solid blue, *A. deanei*. (*B–D*) U walk for different codon positions. (*E*) dN − dS value for each codon position. Values higher than 10 or lower than −10 were cut at such values. Red bars, *P* < 0.05 according to FEL method; blue bars, nonsignificant sites. Values below 0 and with *P* < 0.05 indicate sites under purifying selection.

*COX3*, *ATP6*, and *NAD7* mRNAs are pan-edited in *Trypanosoma* sp., but other trypanosomatids have partially or completely lost editing by replacing the gene by retroposition of the fully edited mRNA (Speijer 2010). Since *T. brucei* shares pan-editing with the ancestor of other kinetoplastids, it can be assumed that most of the amino acid sequence in such an ancestor was similar to *T. brucei* sequence. Consequently, a comparative analysis between *T. brucei* (ancestral) and kinetoplastids that lost pan-editing (derived) may help to infer and understand the changes in hydrophobicity after losing editing and whether these changes had an evolutionary role. Hydrophobicity along protein sequence was highly conserved in *Leishmania tarentolae*, *Crithidia fasciculata*, and *Leptomonas pyrrhocoris* (nonedited or minimally edited) in relation to *T. brucei* (pan-edited) in *COX3* (fig. 6*A*), *ATP6*, and *NAD7* (supplementary fig. S6, Supplementary Material online). In addition, hydrophobicity of COX3 in the kinetoplastid *Angomonas deanei* was mainly conserved with few exceptions near the amino acids 70 and 200 in the sequence (fig. 6*A*, blue line). In order to analyze if such conserved hydrophobicity was caused by purifying selection, we first compared U content across codon positions among kinetoplastid species (fig. 6*B*–*D*). The U content in the second codon position was less variable than that in the first and third ones (fig. 6*C*). However, as mentioned above, protein hydrophobicity in kinetoplastids that lost editing was almost identical to the observed in *T. brucei* proteins. These results suggest a lower substitution rate in the second codon position as expected under purifying selection.

Moreover, we performed a synonymous/nonsynonymous substitutions analysis of *COX3* mRNA sequences from different kinetoplastids. An increase in frequency of synonymous substitutions versus nonsynonymous ones would suggest purifying selection. The analysis showed that 197/288 codons exhibited a site-specific synonymous substitution rate (dS) higher than their nonsynonymous substitution rate (dN), indicating that dN − dS < 0. Of these, 144 codons coded for hydrophobic amino acids in *T. brucei*. Among codons with dN − dS < 0, 126 were found to be significant using the fixed effects likelihood (FEL) method (*P* < 0.05) (see fig. 6*E*). On the other hand, only 40/288 codons had dN − dS > 0, but they were nonsignificant (*P* > 0.1), suggesting neutral evolution instead of positive selection (diversifying selection) (see fig. 6*E*). Most implied hydrophobic-to-hydrophobic or hydrophilic-to-hydrophilic substitutions and just nine codons implied hydrophobic-to-hydrophilic substitutions. All these results suggest purifying selection preventing hydrophobicity loss.

### A Hydrophobic Ratchet Prevents Hydrophobicity Reduction after Editing Loss

**Fig. 7.**
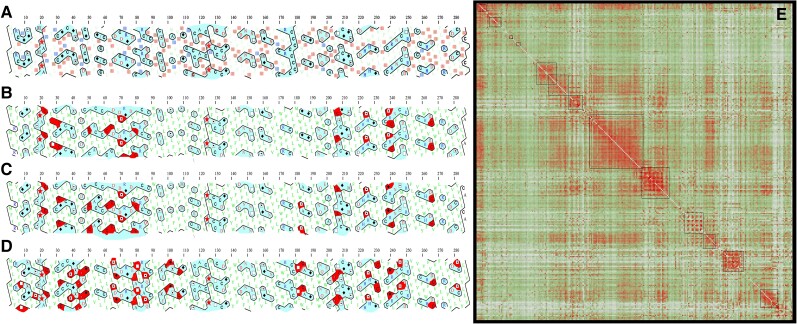
Restricted hydrophobicity loss in COX3 after losing pan-editing. (*A*) HCA for *T. brucei*. Light blue regions represent hydrophilic clusters, whereas pink squares highlight amino acids under purifying selection (*P* < 0.05), and blue squares indicate amino acids that show substitutions by hydrophobic ones in other kinetoplastids that have lost editing in *COX3*. (*B*) HCA for *L. tarentolae* highlighting in red hydrophilic amino acid positions that are hydrophobic in *T. brucei*. (*C*) HCA for *C. fasciculata*. (*D*) HCA for *A. deanei*. (*E*) Heatmap showing COX3 correlation of evolutionary changes between different amino acids by using Coeviz2. The scale goes from green = 0 (low correlation) to red = 1 (high correlation). Red squares on the diagonal of the heatmap are expected for local coevolution of the amino acids.

We performed HCA of protein structures to address where hydrophobic-to-hydrophilic substitutions (hydrophobicity reduction) occurred after editing was lost. Firstly, COX3 protein structure was analyzed and compared among *T. brucei* (pan-edited), *L. tarentolae* (5′ edited), *Crithidia fasciculata*, and *A. deanei* (nonedited). Results showed that whole hydrophobic-to-hydrophilic substitutions occurred at the edge of hydrophilic clusters (fig. 7*A*–*D*). However, sites with strong purifying selection (*P* < 0.05) were also predominantly distributed within and at the edge of hydrophilic clusters. This suggested that such hydrophilic clusters cannot increase in size, at least not by changing only one amino acid at a time (fig. 7*A*). Similar patterns were observed for ATP6 and NAD7 (supplementary figs. S7 and S8, Supplementary Material online). These results may suggest that replacing a single hydrophobic amino acid may be destabilizing for the protein. In fact, multiple substitutions occurring at a time or in short periods and changing two or more amino acids would be required to reduce hydrophobicity without protein destabilization.

It is known that editing can introduce multiple Us in contiguous or close positions, leading to the introduction of multiple hydrophobic amino acids at once. Many times, when two or more interacting hydrophilic amino acids are replaced by hydrophobic ones, the interactions (although of a different type) and the protein structure are preserved. These changes cannot occur step by step because replacing only one hydrophilic amino acid (by a hydrophobic one) breaks the interactions with other hydrophilic residues and destabilize the protein. This may explain why editing may force increased hydrophobicity avoiding deleterious states. Consequently, strong correlations between substitutions of nearby amino acids in the sequence should be observed. To analyze this hypothesis, we used Coeviz2 in order to detect sites that coevolve in COX3 and NAD7 and to analyze if such sites clustered closely together in the amino acid sequence. Figure 7*E* shows a heatmap of sites that coevolved for COX3 (red) and shows clear clusters for closely located amino acids. Finally, it was addressed in the heatmap whether hydrophobic amino acids coevolved. A total of 324 site pairs, located within a five amino acid distance in the sequence, scored >0.8 in the coevolution heatmap. These pairs are located along the diagonal of the heatmap. Among these pairs, 52% consisted of interactions between two hydrophobic amino acids. These results suggest that a hydrophobic ratchet prevents protein hydrophobicity reduction after editing loss.

### Hydrophobicity Increase Was Favored at the Protein Surface

A Blast search was performed for *T. brucei* NAD7, NAD9, ATP6, and COX3 proteins against human protein databases. These analyses were performed against humans since respiratory complexes are well described for this species. NAD7 was the most conserved protein (table 4), and it is homologous to the NADH dehydrogenase (ubiquinone) iron–sulfur protein 2, which was considered in a cryo-EM 3D model of the respiratory complex I (accession: 5XTD) as the chain Q. The solvent-accessible surface area (SASA) of the structures of chain Q and NAD7 was compared. The surface area of NAD7 had an overrepresentation of hydrophobic amino acids (Phe and Leu) and an underrepresentation of hydrophilic ones compared with chain Q surface (supplementary fig. S9, Supplementary Material online). In addition, 44.9% of the exposed sites of NAD7 had higher hydrophobicity than chain Q, while only 20.4% had less hydrophobicity. Furthermore, the sites with higher hydrophobicity in NAD7 than in chain Q were mainly located on the protein surface (44.9% in exposed sites vs. 31.8% in nonexposed sites, *P* = 0.01). Similar results were observed by comparing *T. brucei* and *D. ambulator* NAD7 proteins (40.9% of sites had increased hydrophobicity in exposed sites vs. 28.0% in nonexposed sites, *P* = 0.009). However, the editing frequency of codons that correspond to exposed amino acids was similar to that of nonexposed amino acids (both 46%).

**Table 4.** Search for Homologs of Proteins Coded by Pan-Edited Genes in *T. brucei*.

	*E* value^a^	Identities	Coverage	Positive^e^
NAD7^b^	0.0	134/385 (35%)	96%	194/385 (50%)
COX3^c^	1e^−68^	44/203 (22%)	67%	69/203 (34%)
NAD9^d^	4e^−23^	19/54 (35%)	27%	29/54 (54%)
ATP6	Nonsignificant			
RPS12	Nonsignificant			

a Search was made by Delta-Blast using the protein sequence of *T. brucei* against databases containing only human proteins with default parameters.

b Best match: NADH dehydrogenase ubiquinone iron–sulfur protein 2 (5XTD_Q).

c Best match: cytochrome c oxidase subunit III (AAP90927.1).

d Best match: NADH dehydrogenase ubiquinone iron–sulfur protein 2 (NP_004542.1).

e Sites with a positive score in the alignment.

### Protein–Protein Contact Surfaces May Limit Hydrophobicity Increase and Constrain Editing

Hydrophobicity changes at the protein–protein contact surface were analyzed by searching *T. brucei* homologs of human subunits that interact with chain Q in the complex I. Four proteins were identified by Blast search (table 5). Particularly, two proteins (chains B and P in the 5XTD model) were mitochondrion-encoded in *T. brucei*, and their mRNAs were pan-edited (called *NAD8* and *NAD9*). Around 44% (52/119) sites of chain Q which interact with chains B and P were more hydrophobic in NAD7 than in chain Q. In addition, around 54% (29/54) and 47% (17/36) in chains P and B respectively were also more hydrophobic in the *T. brucei* homologs. This may imply compensatory changes (an example is observed in supplementary fig. S11, Supplementary Material online). However, it cannot be distinguished whether these changes in hydrophobicity are related to interactions with NAD7 or to the editing in the mRNA of such proteins (although both hypotheses are not mutually exclusive). The other two Q-interacting proteins were nuclear-encoded (chains C and M in the 5XTD model of the respiratory complex I). The sites located at the contact surface between chains Q and M were poorly conserved in NAD7 and located in an INDEL region. Instead, the sites located at the interacting surface between NAD7 and *T. brucei* homologs to the chain C were better conserved (supplementary fig. S10, Supplementary Material online). Consequently, we addressed hydrophobicity changes at the Q–C interacting surface in *T. brucei* homologs. The Q–C interface in *T. brucei* NAD7 had less hydrophobicity increase —calculated as the percent of amino acids with higher hydrophobicity—compared with sites outside such interacting surface on the same protein (17% vs. 44%, *P* = 0.0008). In addition, the contact surface to NAD7 in the *T. brucei* chain C homolog had a hydrophobicity increase not higher than that observed for other sites in the protein (18.9% vs. 28.5%, *P* = 0.24). In addition, the NAD7 codons that code for the Q–C interface were significantly less edited than other sites in mRNA (25.2% vs. 47.4%, *P* = 3 × 10^−6^). This result shows that this interacting surface may limit the extent of hydrophobicity increase in the NAD7 protein and constrain editing of its mRNA.

**Table 5.** Search for *T. brucei* Homologs of the Proteins that Interacts with Chain Q in Model 5XTD of the Human Respiratory Complex I.

Chain	Best match^a^	Identities	Coverage	*E* value
P	NAD9 (AAA03749.1)^b^	16/40 (40%)	19%	5e−5
B	NAD8 (P30826.1)	38/87 (44%)	49%	8e−17
M	electron transfer protein, putative (XP_011778675.1)	84/204 (41%)	28%	2e−80
C	NADH-ubiquinone oxidoreductase 20-kDa subunit (XP_011779287.1)	87/147 (59%)	94%	5e−73

a Best match for *T. brucei*.

b Accession number in NCBI protein.

## Discussion

Since the discovery of kinetoplastids mRNA editing system in the mitochondrion in the 1980s (Benne et al. 1986; Feagin et al. 1988; Shaw et al. 1988), several questions remain, or at least, are incompletely answered. Why is such an energetically expensive system for editing a dozen (or fewer) mitochondrion-encoded genes maintained? Why did natural selection not erase such complex machinery? This is more disconcerting if we consider that kDNA involves around or more than 50% of the total cellular DNA in some kinetoplastids such as *Perkinsela sp.* or *Trypanoplasma borreli* (Lukes et al. 2018). Several attempts to answer such questions were made. These hypotheses range from a simple mutation correcting system (Benne et al. 1986; Gray 2003), an expression regulation method (Shaw et al. 1988), a mutational machinery to generate protein diversity (Landweber and Gilbert 1993) and alternative functions (Ochsenreiter et al. 2008), a mechanism to protect genes from deletions in complex life cycles (Speijer 2006, 2008), a machinery to generate DNA/RNA hybrids that promotes transcription termination and DNA replication (Leeder et al. 2016) to a dead-end moved by an evolutionary neutral ratchet (Gray et al. 2010; Flegontov et al. 2011), or a combination of different ones (Lukes et al. 2021).

Another big question is how the transition from nonedited genes to pan-edited ones occurred. In this matter, a three-step model was proposed (Covello and Gray 1993). First, the RNA editing capability appeared. Second, mutations that could be corrected occurred, and they were then fixed by genetic drift. Third, natural selection conserved RNA editing (Covello and Gray 1993). gRNAs directing the editing appeared later. Here, we contribute to the understanding of the increase in editing, resulting in pan-edited genes. Furthermore, we described some basic features of mRNA editing considering its effect on the coding sequence and on the protein. Our results clearly showed that mRNA editing in kinetoplastids mainly introduced Us with no codon position preference. However, such editing favored hydrophobic amino acids and hydrophobic regions in the protein by inserting Us at second codon position. The hydrophobic increase was distributed throughout the protein sequence, with a slightly greater preference for protein surface, at least in NAD7 (such surface preference was not observed for editing). However, protein–protein interactions between NAD7 and a nuclear-encoded protein may have influenced where editing occurred by limiting the hydrophobicity increase. In contrast, the contact surfaces between proteins that can be edited may have undergone compensatory changes that increased their hydrophobicity. Such changes may have been facilitated by editing as observed in the interactions between NAD7 and NAD8 or NAD9. Moreover, after editing was lost by retroposition in some kinetoplastids (Simpson and Maslov 1994b), loss of hydrophobicity was prevented by purifying selection, which is indicated by a lower substitution rate on the second codon position. Only a few substitutions from hydrophobic amino acids to hydrophilic ones were observed, and they occurred spatially close to other hydrophilic residues. And indeed, the analysis of coevolving sites clearly showed many local patterns suggesting that multiple mutations at a time are required for hydrophobicity loss around the hydrophilic clusters. Altogether, these findings suggest that a ratchet may be preventing hydrophobicity reduction; that is, multiple mutations at a time are required for variations in hydrophobicity.

Based on such results, we propose that editing did not occur at random positions in the mRNA. Instead, protein hydrophobicity influenced RNA editing site selection and where editing could be expanded. At the protein level, editing was probably allowed within or at the edge of hydrophobic clusters of amino acids. Additionally, it often involved multiple substitutions of closely situated amino acids simultaneously. If editing was lost by retroposition of the fully edited mRNAs, the changes made by editing could not be easily reverted by single-point mutations because intermediate states are deleterious. Consequently, they entrenched more and more hydrophobicity in the protein. We propose the following scenario for the evolution toward pan-editing. In the kinetoplastid ancestor, eventual deletions of one or few nucleotides occurred within maxicircles coding regions. If those deletions could be corrected, one or few Us would be introduced in the mRNA. Such mRNA editing can be compared with a mutational pressure to introduce Ts in DNA, like in ctenophores (see fig. 5 and Wang and Cheng 2019). However, changes in the amino acid properties in the translated protein generate constraints to the sites where editing can insert Us. This is caused by certain spatial clustering of hydrophobic (or order-promoting) amino acids. Consequently, editing is allowed within and at the edge of such clusters, whereas in other cases, inserting multiple Us at once was necessary to preserve protein stability. Interestingly, as microdeletions in the coding regions may simultaneously affect several codons, mRNA editing could correct deletions of amino acids that were spatially neighbors. Despite this, such multiple codon modifications did not necessarily occur at the same time, particularly if purifying selection is not so strong (Lovell and Robertson 2010). It is probable that changes in hydrophobicity could be slow at the beginning, but as more and more hydrophilic amino acids were replaced by hydrophobic ones, later changes could be less destabilizing, and consequently, the process could speed up.

In addition, we propose that natural selection does not appear to play a role in favoring an increase in hydrophobicity, as hydrophobicity can affect various regions of proteins and different types of proteins (such as transmembrane and soluble proteins from respiratory complexes or elsewhere, such as RPS12) (Opperdoes and Michels 2008; Aphasizheva et al. 2013) and editing seemingly occurred wherever it was possible. In addition, editing affects any codon position. A neutral hydrophobic ratchet has been previously proposed for explaining the evolution of multimeric proteins (Hochberg et al. 2020), and it may also apply to intramolecular amino acid composition. Some mutational pressures, for example, mutational bias in ctenophores (Wang and Cheng 2019) or editing in kinetoplastids, not only influenced amino acid composition but we propose they may also have ratcheted it. Anyway, protein function is conserved revealing how flexible amino acid sequences are.

It is unclear why editing was evolutionarily preserved in many genes and many lineages considering the possibility of retroposition of the fully edited mRNA. Complex systems or energetically unfavorable mechanisms, according to the CNE hypothesis, are only conserved if it is highly unlikely to reach the previous state (a ratchet) (Gray et al. 2010; Hochberg et al. 2020). One possibility is that new essential functions have appeared (Lukes et al. 2021). Alternatively, a new hypothesis may be derived from our results. Basically, loss of editing may be unfavorable because the proteins are entrenched in a hydrophobic extreme and mutational pressure tends to reverse the high T content, making most nonsynonymous mutations deleterious. Still, more testing is required.

Alternatively, it is important to note that loss of editing due to gene replacement should leave the gene susceptible to be reedited. There are two possible reasons for this hypothesis: first, the poly-T regions are very susceptible to indels because of polymerase slippage. Second, the hydrophobic ratchet maintains the sequence easily editable; that is, new deletions probably do not imply that editing replaces hydrophilic amino acids by hydrophobic ones. Consequently, editing loss and restoration are expected to be part of a dynamic process. However, it is unclear whether such a process occurs.

Finally, plastid mRNA editing in other organisms such as plants has also been reported to generate substitutions that favor hydrophobicity in the protein and are related to the restoration of functional domains as alpha-helices (Jobson and Qiu 2008; Yura and Go 2008; He et al. 2016; Koo and Yang 2020). In consequence, could hydrophobic constraints (or requirements) be the link between very different and evolutionarily unrelated mechanisms such as editing in plants and editing in kinetoplastids? Further analyses in more species, sequencing of mitochondrial genomes and functional analyses may help answer such question. In conclusion, based on our results, we propose a mechanism for a transition toward pan-edited mRNA in ancestral kinetoplastids directed by hydrophobic constraints in the protein structure and suggest a new hypothesis about the evolution of this complex system.

## Materials and Methods

### Sequences

The following mitochondrion-encoded sequences (genes or fully edited mRNAs): *COX1*, *COX2*, *COX3*, *CYTB*, *NAD1*, *NAD5*, *NAD7*, *NAD9*, *RPS12*, and *ATP6* were downloaded from GenBank for kinetoplastids (nine species), diplonemids (two species), euglenids (one species), and jakobids (two species). A total of 119 sequences were analyzed. The different species and accession numbers are provided in supplementary material S1, Supplementary Material online. Noncoding regions were trimmed. Additional protein sequences of cytochrome c oxidase subunit III (COX3) were downloaded from the RefSeq database of NCBI (supplementary material S2, Supplementary Material online).

### DNA–RNA Sequence Analysis

U content across the mRNA sequence was addressed by a unidimensional U walk (Berger et al. 2004). For a position *i* in the mRNA sequence, define the value x[i] = +1 if a U is present or the value x[i] = −1 if another nucleotide is present. Consequently, for each position it can be defined $s[U_{k}]$ as:


$$s[U_{k}]=\overset{k}{\underset{i=1}{\sum }}\;x[i]\;$$

A line is used to represent the values showing, for each position, an increase in one unit whenever there is a U and a decrease in one unit when there is another nucleotide. Consequently, it is possible to address both the overall U content (last point of the line) and U content at different regions, in the same graph. Positive slopes indicate more Us than other bases. Regions with the same slopes (parallels) indicate similar U content. The approach was used to address U content at each codon position. In addition, the standard deviation for the U content along every sequence in each codon position was determined using a nonoverlapped sliding window of 30 bases. The hypothesis of uniform distribution of the U content at different codon positions was evaluated by using a chi-square test. In addition, the hypothesis of uniform distribution of inserted Us at different codon positions was also evaluated. Inserted Us were inferred by a manual comparison between the fully edited mRNAs and the corresponding cryptogenes. In order to address purifying, neutral, and diversifying selection, the site-specific nonsynonymous and synonymous (dN and dS, respectively) substitution rates were inferred by using FEL (Kosakovsky Pond and Frost 2005) in the Datamonkey server (Weaver et al. 2018). The FEL method utilizes a maximum-likelihood approach to estimate the rates of nonsynonymous (dN) and synonymous (dS) substitutions at each site in a coding alignment and its phylogeny. Then, a likelihood ratio test is used to test if dN is significantly different to dS (Kosakovsky Pond and Frost 2005).

### Protein Sequence Analysis

Hydrophobicity of different amino acid sequences was evaluated by using the Kyte–Doolittle index (Kyte and Doolittle 1982). The cumulative hydrophobicity was graphed in order to compare against U walks. Amino acid composition was estimated by using MEGA v7 (Kumar et al. 2016). Amino acids were classified as order—and disorder—promoting according to the criteria in Dunker et al. (2001). HCA (Gaboriaud et al. 1987; Woodcock et al. 1992; Bitard-Feildel et al. 2018; Lamiable et al. 2019) was made using the DrawHCA tool (http://osbornite.impmc.upmc.fr/hca/hca-form.html). Search for homologous proteins of humans was made by Delta-Blast search.

### Protein Structure Analysis

Prediction of the protein structure was made using the RoseTTAFold server (https://robetta.bakerlab.org/submit.php) (Baek et al. 2021), and the structure was drawn with the software ChimeraX v1.2.5 (Pettersen et al. 2021). Coevolving sites were analyzed by using CoeViz2 (Corcoran et al. 2021) with a 20-amino acid alphabet, and the Uniprot-Uniref90 database to build the multiple sequence alignment. Surface-exposed amino acids in the above structures and in protein structures of the complex I downloaded from NCBI Protein (accession: 5XTD) were calculated by determining the SASA using the server of the Center for Informational Biology (http://cib.cf.ocha.ac.jp/bitool/ASA/). Amino acid positions exposed to the solvent were defined as those with an exposed area higher than 30%. Also, contact surface sites between two proteins in the 3D model 5XTD of the respiratory complex were studied. Contact surfaces were determined by comparing SASA for two proteins in the complex I individually, against their SASA when such proteins are interacting according the 5XTD model. Sites that evidenced any reduction in the SASA value when proteins interact were considered to be located in surface contact areas. Hydrophobicity increases were determined by using the Kyte–Doolittle index. The number of sites for which hydrophobicity was higher in *T. brucei* proteins than in their human homologs was calculated for exposed, nonexposed, interacting, and noninteracting sites. They were compared by using a chi-square test.

## Supplementary Material

msad081_Supplementary_Data

## Data Availability

All the sequences used in this manuscript were obtained from public databases and the accession numbers are provided in File S1.
