# Supplementary material for: Hydrophobicity-Driven Increases in Editing in Mitochondrial mRNAs during the Evolution of Kinetoplastids
Source: Mol Biol Evol. 2023 Apr 8;40(4):msad081. doi: 10.1093/molbev/msad081 (PMC10118304; doi:10.1093/molbev/msad081)
Supplement: msad081_Supplementary_Data [file msad081_supplementary_data.zip › Supplementary Figures.pdf]

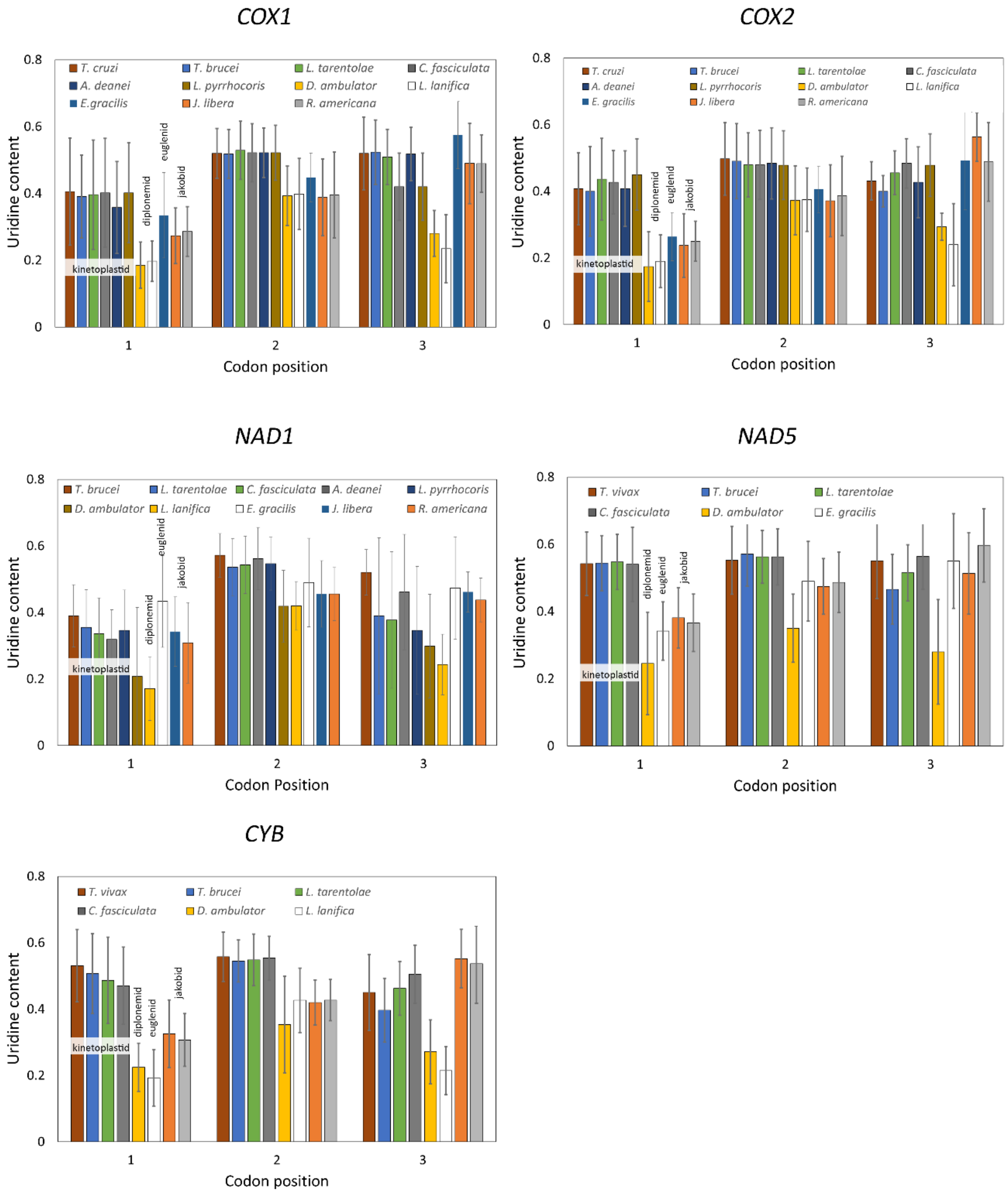

Figure S1. Uridine content in different codon positions for genes partially or non-edited in *Trypanosoma brucei* and related groups with available sequences.

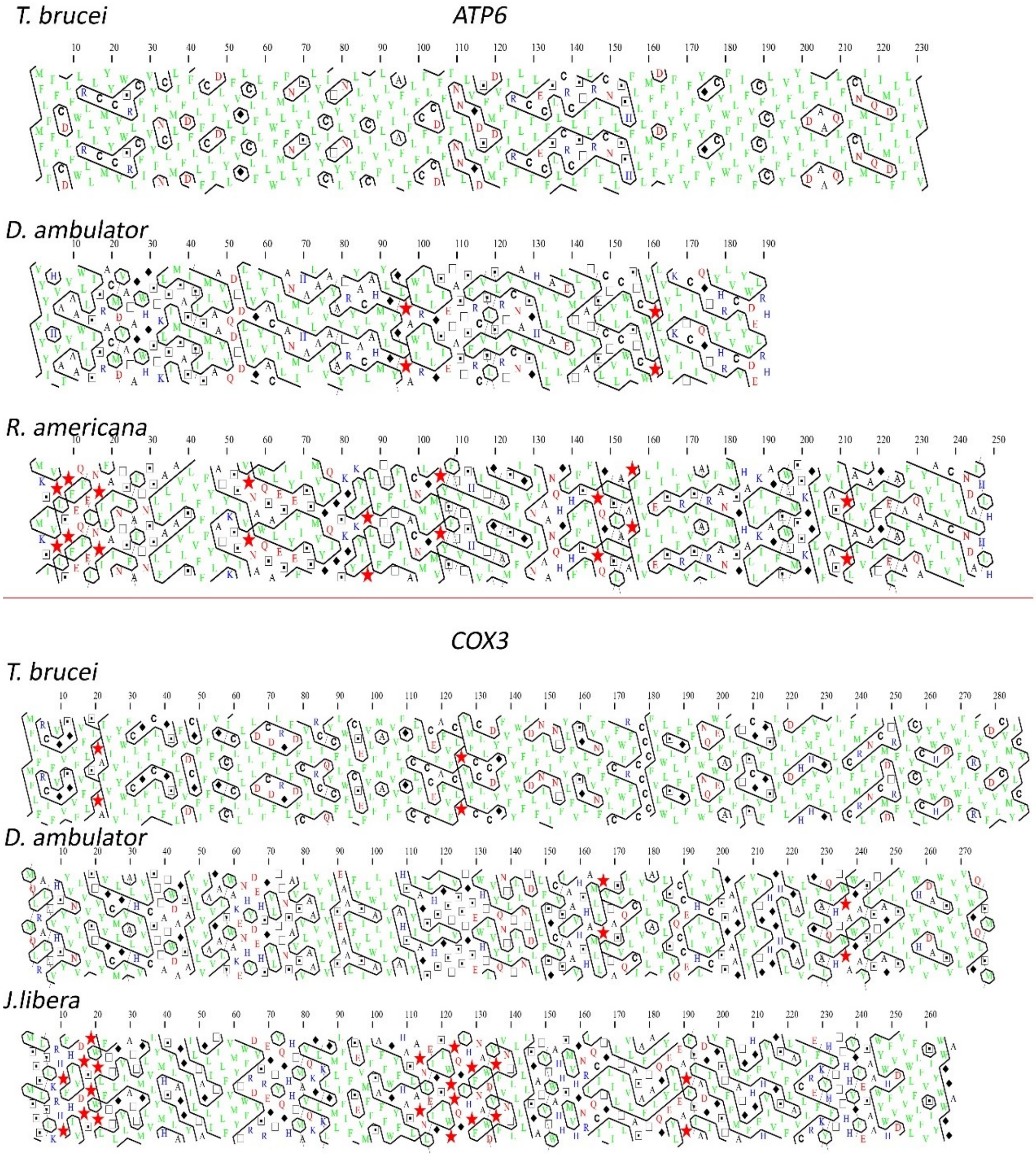

Figure S2. Hydrophobic cluster analysis of ATPase subunit 6 (ATP6) and Cytochrome c Oxidase subunit 3 (COX3)

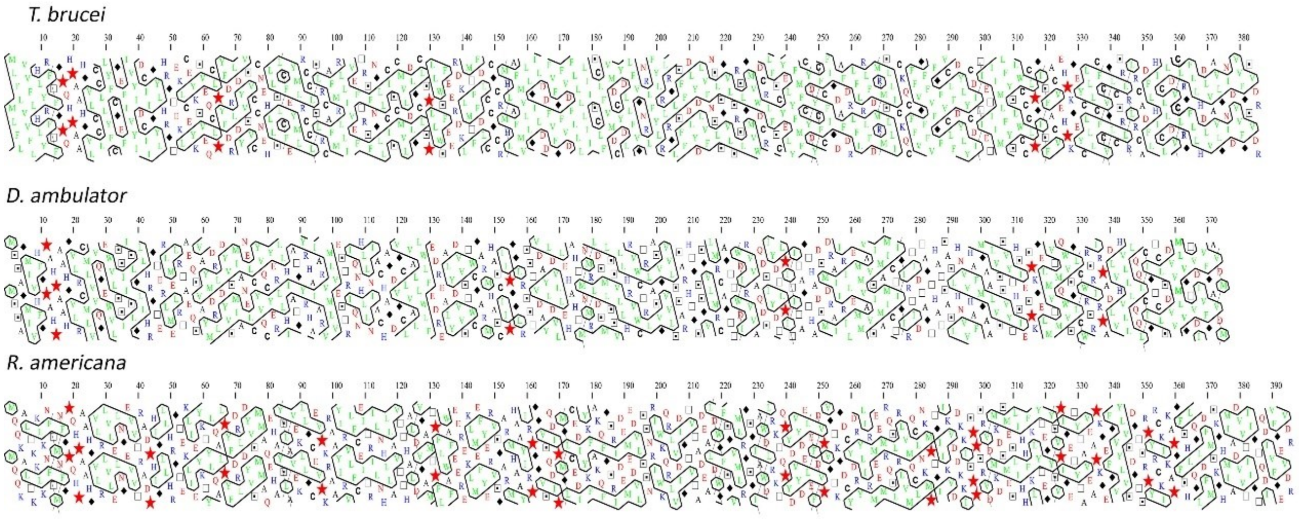

Figure S3. Hydrophobic cluster analysis of NAD dehydrogenase subunit 7 (NAD7)

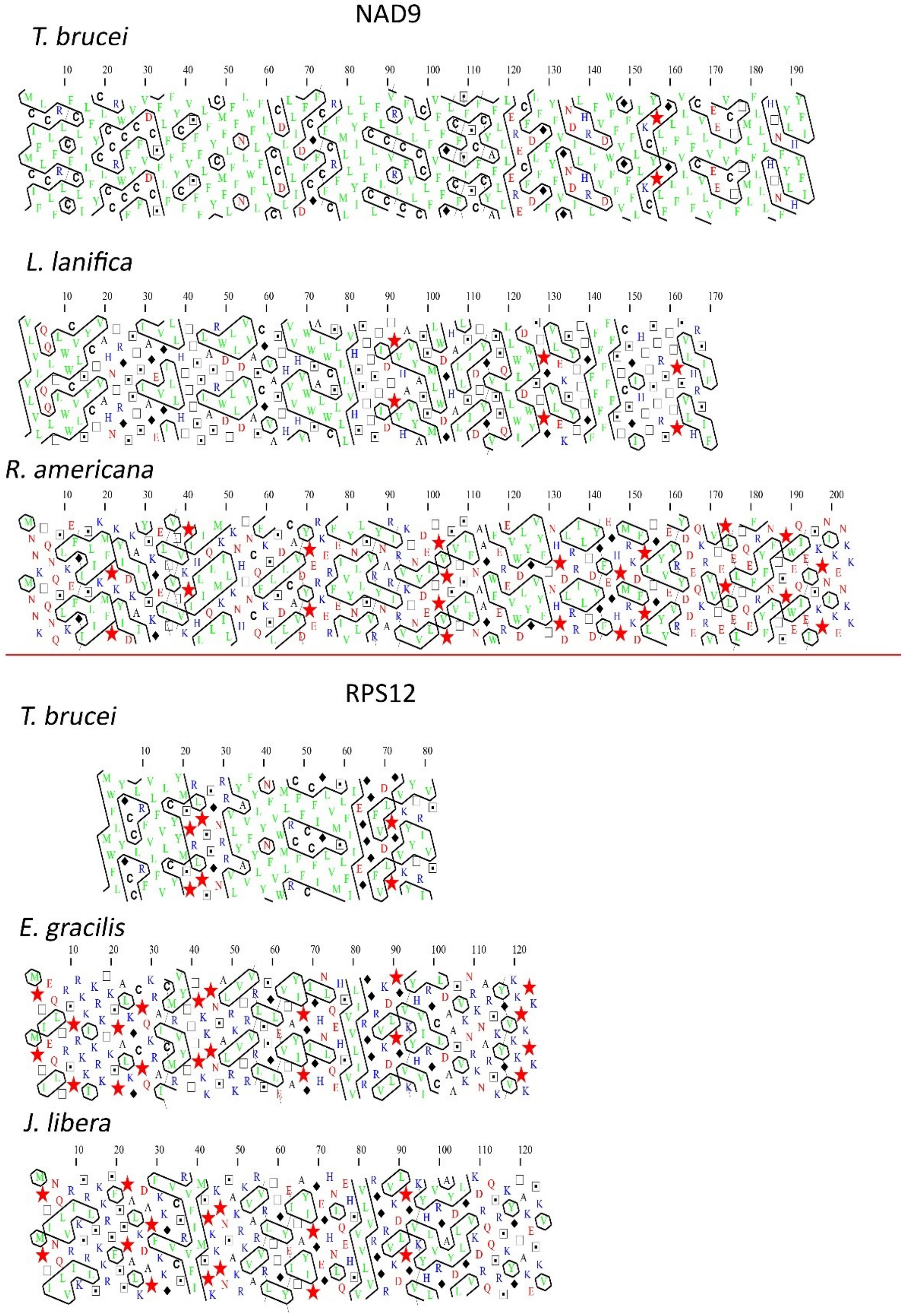

Figure S4. Hydrophobic cluster analysis of NAD dehydrogenase subunit 9 (NAD9) and Ribosomal protein S12 (RPS12)

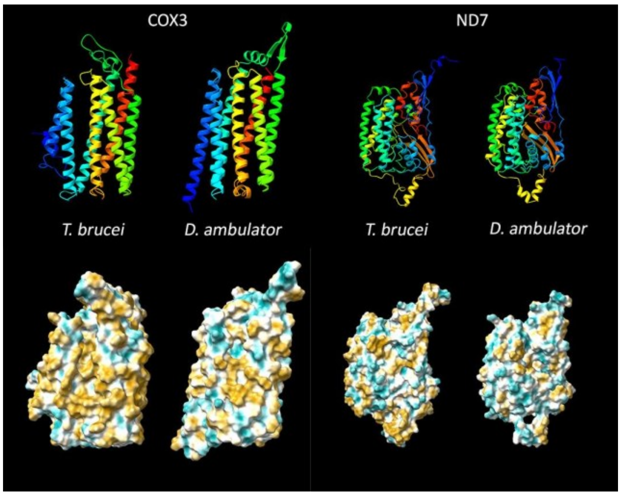

Figure S5. Protein structure prediction of COX3 and NAD7 from *Trypanosoma brucei* and *Diplonema ambulator*. Up, cartoon representation of alpha-helices, beta-strands, and disordered regions predicted by RoseTTAFold. Down, surface hydrophobicity coloring ranging from dark cyan (most hydrophilic) to white to dark golden (most hydrophobic) regions calculated on ChimeraX.

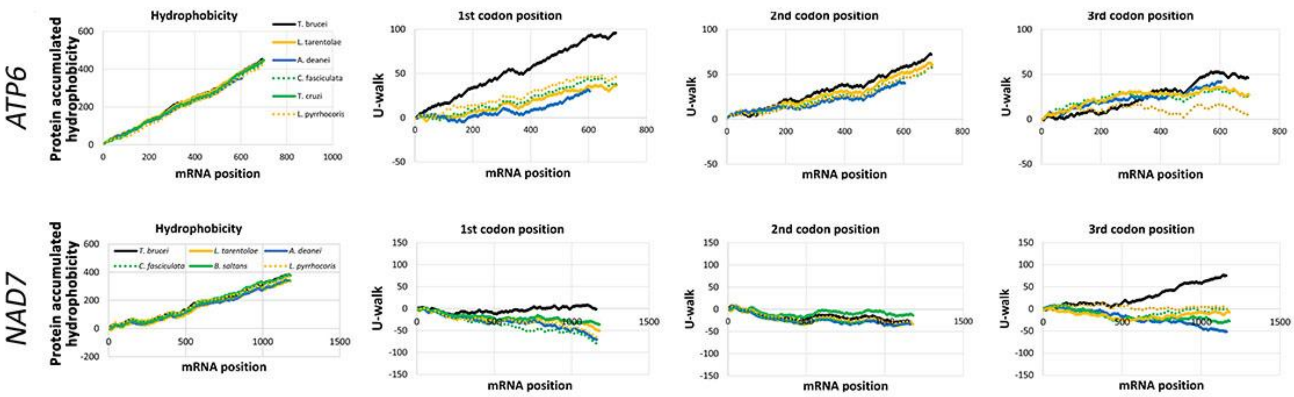

Figure S6. Cumulative hydrophobicity and U-walks for kinetoplastids

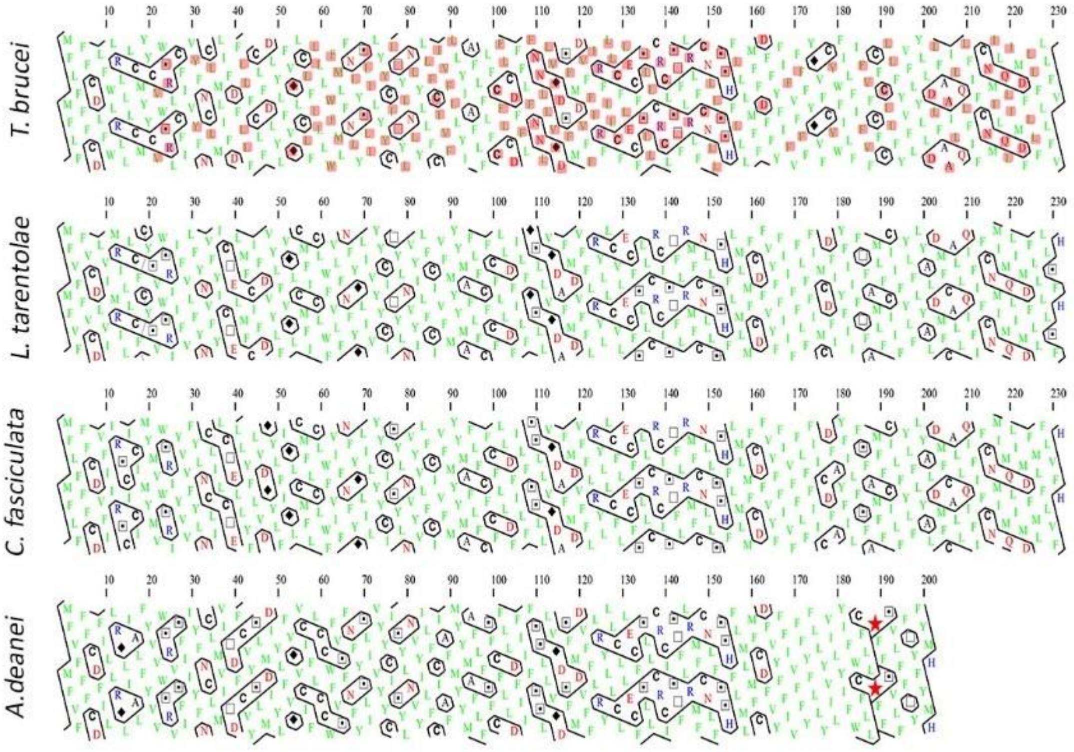

Figure S7. Hydrophobic clusters for ATP6 in different kinetoplastids

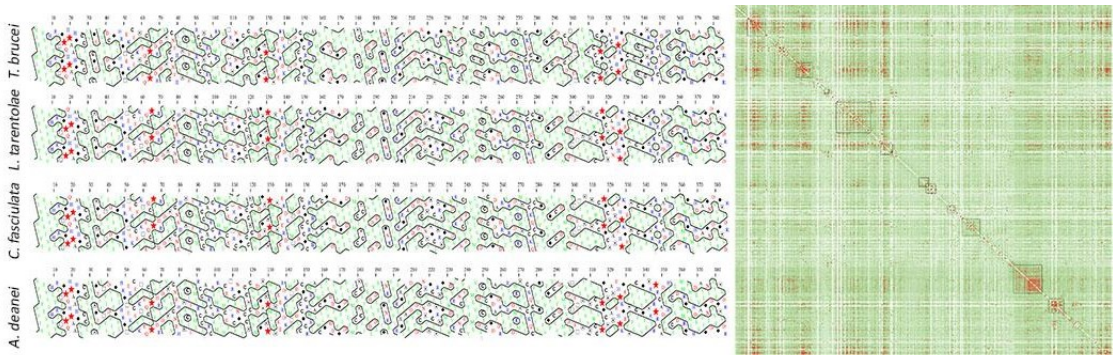

Figure S8. Hydrophobic clusters for NAD7 in different kinetoplastids (left) and heatmap showing coevolutionary substitutions for different sites

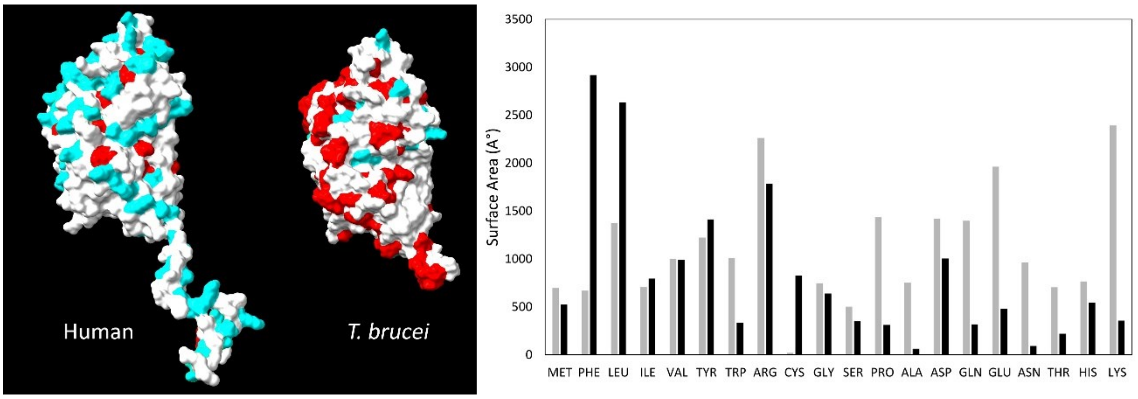

Figure S9. Increased content of hydrophobic amino acids at NAD7 surface compared to the human counterpart. Left, surface content of Leu and Phe (red, hydrophobic) and Pro, Gln, Glu, Asn (cyan, hydrophilic). Right, solvent accessible surface area of each NAD7 amino acid in human (gray) and *T. brucei* (black).

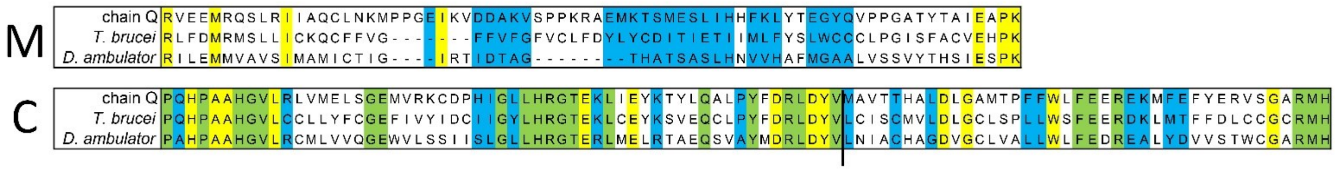

Figure S10. Conserved regions in the chain Q (human) and NAD7 (*T. brucei* and *D. ambulator*) in the contact surface to chains M and C. Yellow, conserved sites near the contact surface. Green, conserved sites located on the contact surface. Blue, non-conserved sites located on the contact surface. Black vertical line indicates non-contiguous sites on the primary structure.

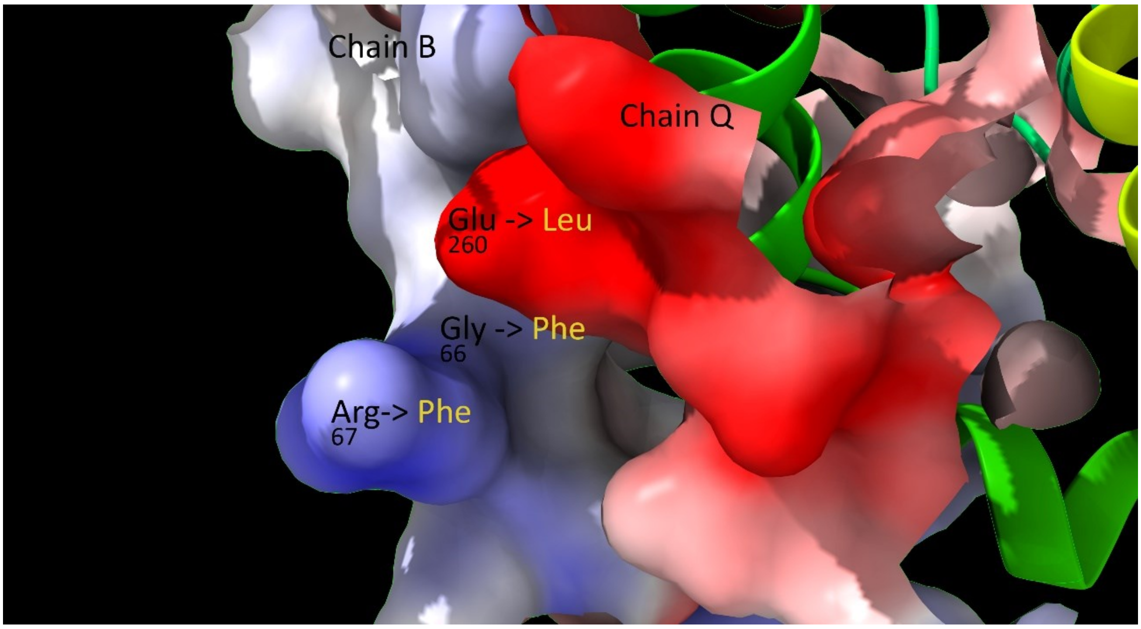

File S11. Possible compensatory change in the interacting surface between chain Q (NAD7 homologue) and chain B (NAD8 homolog) of the respiratory complex I (accession 5XTD). The surface is colored according to an electrostatic scale (blue = positively charged, red= negatively charged). Note that the highlighted amino acids positions correspond to hydrophobic amino acids in the NAD7 and NAD8 proteins of *T. brucei* suggesting the replacement of electrostatic interaction by hydrophobic ones.
